# The use of the integrated cognitive assessment to improve the efficiency of primary care referrals to memory services in the accelerating dementia pathway technologies study

**DOI:** 10.3389/fnagi.2023.1243316

**Published:** 2023-09-13

**Authors:** Mohammad Hadi Modarres, Chris Kalafatis, Panos Apostolou, Naji Tabet, Seyed-Mahdi Khaligh-Razavi

**Affiliations:** ^1^Cognetivity Ltd., London, United Kingdom; ^2^South London & Maudsley NHS Foundation Trust, Department of Old Age Psychiatry, King’s College London, London, United Kingdom; ^3^Centre for Dementia Studies, Brighton & Sussex Medical School, Brighton, United Kingdom; ^4^Department of Stem Cells and Developmental Biology, Cell Science Research Centre, Royan Institute for Stem Cell Biology and Technology, ACECR, Tehran, Iran

**Keywords:** ICA, CognICA, AI, Alzheimer disease, cognitive assessment, health economics, ADePT

## Abstract

**Background:**

Current primary care cognitive assessment tools are either crude or time-consuming instruments that can only detect cognitive impairment when it is well established. This leads to unnecessary or late referrals to memory services, by which time the disease may have already progressed into more severe stages. Due to the COVID-19 pandemic, some memory services have adapted to the new environment by shifting to remote assessments of patients to meet service user demand. However, the use of remote cognitive assessments has been inconsistent, and there has been little evaluation of the outcome of such a change in clinical practice. Emerging research has highlighted computerized cognitive tests, such as the Integrated Cognitive Assessment (ICA), as the leading candidates for adoption in clinical practice. This is true both during the pandemic and in the post-COVID-19 era as part of healthcare innovation.

**Objectives:**

The Accelerating Dementias Pathways Technologies (ADePT) Study was initiated in order to address this challenge and develop a real-world evidence basis to support the adoption of ICA as an inexpensive screening tool for the detection of cognitive impairment and improving the efficiency of the dementia care pathway.

**Methods:**

Ninety-nine patients aged 55–90 who have been referred to a memory clinic by a general practitioner (GP) were recruited. Participants completed the ICA either at home or in the clinic along with medical history and usability questionnaires. The GP referral and ICA outcome were compared with the specialist diagnosis obtained at the memory clinic.

Participants were given the option to carry out a retest visit where they were again given the chance to take the ICA test either remotely or face-to-face.

**Results:**

The primary outcome of the study compared GP referral with specialist diagnosis of mild cognitive impairment (MCI) and dementia. Of those the GP referred to memory clinics, 78% were necessary referrals, with ~22% unnecessary referrals, or patients who should have been referred to other services as they had disorders other than MCI/dementia. In the same population the ICA was able to correctly identify cognitive impairment in ~90% of patients, with approximately 9% of patients being false negatives. From the subset of unnecessary GP referrals, the ICA classified ~72% of those as not having cognitive impairment, suggesting that these unnecessary referrals may not have been made if the ICA was in use. ICA demonstrated a sensitivity of 93% for dementia and 83% for MCI, with a specificity of 80% for both conditions in detecting cognitive impairment. Additionally, the test-retest prediction agreement for the ICA was 87.5%.

**Conclusion:**

The results from this study demonstrate the potential of the ICA as a screening tool, which can be used to support accurate referrals from primary care settings, along with the work conducted in memory clinics and in secondary care. The ICA’s sensitivity and specificity in detecting cognitive impairment in MCI surpassed the overall standard of care reported in existing literature.

## Introduction

Worldwide, national dementia strategies emphasize the need for improving the diagnostic pathway at the point of primary care toward timely diagnosis. Currently, General Practitioners (GPs) clinical judgment of cognitive impairment is the basis of referral initiation to specialist services. Existing primary care cognitive assessment tools (GPCOG, Mini-Cog, 6CIT etc.), are crude or time-consuming, screening instruments which can only detect cognitive impairment when it is well established. Dementia is difficult to diagnose; in a study concerning false positive diagnoses, 60% of GPs misdiagnosed dementia (Shinagawa et al., 2016). More detailed tests deployed in secondary-care are expensive and often physically and psychologically intrusive for the patient (e.g., lumbar puncture). As a result, many false-positives are identified in referred patients. A key limitation of existing screening tests is the lack of robust evidence to support them; few have been well validated in the populations which they are intended for.

**Figure 1:**
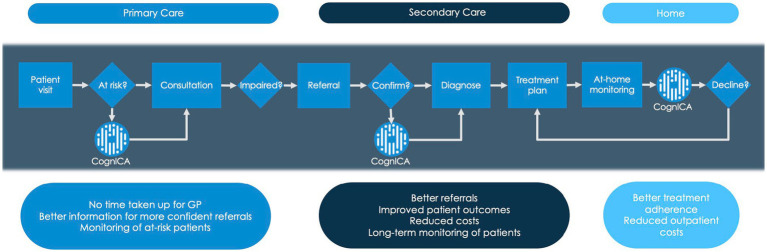
Dementia diagnostic pathway. This figure describes where the ICA tool (i.e., CognICA) can be introduced within the existing dementia pathway.

Figure 1 demonstrates the dementia diagnostic pathway for patients, and highlights places where the ICA tool (i.e., CognICA) can be introduced for better outcome. Patients who are referred by GP are triaged. At the memory clinic patients usually undergo two appointments; the first is typically conducted by a nurse or other non-medical professional and involves administration of a cognitive assessment. At the second appointment (i.e., the diagnostic clinic visit), conducted by a dementia medical specialist, the patient receives the outcome of the assessment (e.g., Diagnosis of dementia, MCI, or Healthy). In light of Covid-19, memory clinics have adapted to the new environment by moving to remote patient assessments in order to continue meeting service user demand while reducing viral transmission. As a result, the majority of appointments were conducted remotely with the use of pen-and-paper tests that are acceptable for remote use.

The COVID-19 pandemic effectively brought clinical practice in the memory services to a standstill. Nationally, memory services adapted to the new environment by moving to remote patient assessments in order to continue meeting service user demand while reducing viral transmission (Owens et al., 2020). However, the remote use of cognitive assessments has been variable, non-standardized while there has been scant evaluation of the outcome of such a change in clinical practice (Binng et al., 2020). Emerging research in remote memory clinics has highlighted computerized cognitive tests such as the ICA as a prominent candidate for adoption in clinical practice both during the pandemic and for post-COVID implementation as part of healthcare innovation (Dunne et al., 2021).

The ICA is a 5-min, self-administered computerized cognitive test based on a rapid categorization task that employs an Artificial Intelligence model to improve its accuracy in detecting cognitive impairment (Kalafatis et al., 2021). The ICA is self-administered and independent of language (Khaligh-Razavi et al., 2019, 2020). The value proposition of the ICA is that a more accurate and sensitive tool for diagnosis will streamline the diagnosis of dementia by reducing false positive results from GP referrals and therefore minimizing the need for further, expensive and time-consuming assessments.

The Accelerating Dementias Pathways Technologies (ADePT) Study was initiated in order to address this challenge and develop a real-world evidence basis to support the adoption of ICA as an inexpensive screening tool for the detection of cognitive impairment and improving the efficiency of the dementia care pathway.

The primary objective for the ADePT study was to deliver real-world evidence on practices and the economic case for ICA adoption in memory clinics for the assessment of cognitive impairment associated with dementia, Alzheimer’s Disease (AD), Mild Cognitive Impairment (MCI), and similar diseases, including assessment of preferred business models by comparing the precision of GP referrals against the ICA.

This report focuses on the results from the clinical study, which is one of the work packages of the ADePT study. The results from this study inform the health economics model (e.g., Shore et al., 2022), which has been delivered separately by York Health Economics Consortium.

## Methods

### The ICA test description

The ICA test (also referred to as Cognetivity’s ICA or **‘CognICA’**) has been described in detail in previous publications (Kalafatis and Khaligh Razavi, 2019; Kalafatis et al., 2019; Khaligh-Razavi et al., 2019; Kalafatis et al., 2021). The ICA test is a rapid visual categorization task. The test takes advantage of the human brain’s strong reaction to animal stimuli. One hundred natural images (50 of animals and 50 of not containing an animal) of various levels of difficulty are selected and are presented to the participant in rapid succession.

Each image is presented for 100 ms followed by a 20 ms inter-stimulus interval (ISI), followed by a dynamic noise mask (for 250 ms), followed by the subject’s categorization into animal vs. non-animal.

### Accuracy, speed, and summary ICA index calculation

The raw data from the ICA is composed of reaction time and categorization accuracy on the images. This data was used to calculate summary features such as overall accuracy, and speed using the same methodology as described previously (Khaligh-Razavi et al., 2019; Kalafatis et al., 2021).

Accuracy is defined as follows:


$$\mathrm{Accuracy}=\frac{\mathrm{Number}\;\mathrm{of}\;\mathrm{correct}\;\mathrm{categoristions}}{\mathrm{Total}\;\mathrm{number}\;\mathrm{of}\;\mathrm{images}}\times 100.$$

Speed is defined based on participant’s response reaction times in trials they responded correctly:


$$\mathrm{Speed}=\min\left[100,100\;e^{-\frac{\mathrm{mean}\;\mathrm{correct}\;RT}{1025}+0.341}\right]\;\text{.}$$

A summary ICA Index, is calculated as follows:


$$ICA\;\mathrm{Index}=\left(\frac{\mathrm{Speed}}{100}\times \frac{\mathrm{Accuracy}}{100}\right)\times 100.$$

The ICA Index describes the raw test result, incorporating speed and accuracy, the two main elements of the ICA test.

### ICA AI model

The AI model utilizes inputs from accuracy and speed of responses to the ICA rapid categorization task (with the ICA Index as an input feature), as well as age, and outputs an indication of likelihood of impairment (AI probability) by comparing the test performance and age of a patient to those previously taken by healthy and cognitively impaired individuals. The AI model is able to achieve an improved classification accuracy relative to using any single feature from the ICA test.

A probability threshold value of 0.5 was used to convert the AI probability to the AI prediction of healthy or cognitively impaired (MCI/mild AD).

The ICA AI model used in this study was a binary logistic regression machine learning model which is a supervised linear classifier. The algorithm’s task is to learn a set of weights from a regression model that maps the participant’s ICA test results and demographics to the classification label of healthy or cognitively impaired.

The ICA AI model was exclusively trained on previous datasets, including data from Khaligh-Razavi et al. (2019) and Kalafatis et al. (2021), and no data from the ADePT study was used to re-train or adjust the model.

### Ethics approval

Health Research Authority and Health and Care Research Wales approval for this study was obtained in February 2020. The study is registered in the ISRCTN Registry (ISRCTN16596456). Approved 27/02/2020, North of Scotland Research Ethics Committee (Summerfield House, 2 Eday Road, Aberdeen, AB15 6RE, UK; +44 (0)1224 558458; nosres@nhs.net), ref: 20/NS/0029.

### Study design

All participants were recruited among attendees at the National Health Service (NHS) memory assessment services at the point of referral by their GP. Participants were recruited from Devon Partnership NHS Trust, North Bristol NHS Foundation Trust, Oxford Health NHS Foundation Trust, and Sussex Partnership NHS Foundation Trust.

Protocol for the Accelerating Dementia Pathway Technologies (ADePT) Study (Kalafatis et al., 2022) is also published and includes more detailed information about the study protocol and procedures.

The participants who did not have a formal diagnosis of a neurodegenerative disease were triaged as per usual clinical practice and were asked to complete the ICA in parallel with the diagnostic assessment.

The main study inclusion criterion was referral to the memory clinic by a GP. Patients recruited were 55 to 90 years old. Potential participants had to be fully informed of and understand the objectives, procedures, and possible benefits and risks of the study and have the capacity to provide written consent.

Subjects that met the following criteria were excluded from the study cohort:

- Lack of capacity to consent to participation in this study
- Upper limb arthropathy or motor dysfunction that limits the use of a tablet computer
- Visual impairment severe enough to limit the use of a tablet computer
- Known diagnosis of dementia
- Already receiving cholinesterase inhibitors and/or Memantine.

### Study procedures

Participants enrolled in the study were required to attend one visit at a designated memory clinic or remotely at their home (Assessment Visit 1 [AV1]). Participants were asked to complete the ICA. Prior to taking the ICA, participants were requested to view a short training video to assist them in completing the task successfully. After taking the ICA, patients completed the following short questionnaires:

- Inquiry on stimulants, fatigue, and sleep: A questionnaire that assesses the participant’s overall state. Questions revolve around recent intake of stimulants (e.g., coffee or alcohol), sleep quality, energy levels, and mood. The questionnaire was used in conjunction with the ICA to determine whether any of these factors have had an impact on ICA performance.
- ICA usability questionnaire: A questionnaire that assesses the participant’s views on their experience with the test to receive acceptability and usability feedback for the ICA.
- Cognitive health questionnaire: A questionnaire that assesses the participant’s history of activities of daily living and physical and mental health comorbidities. The questions should ideally be answered by the informant (study partner) if available or by the participant if an informant is not present. The questionnaire was used in conjunction with the ICA to determine whether cognitive impairment detected by the ICA was due to MCI/dementia or other organic and/or treatable conditions. The results from the cognitive health questionnaire are shown in Appendix A3.

Lastly, a brief medical history of the participants via electronic health care records was obtained, mainly focusing on any cognitive tests that have been taken by the participants.

**Figure 2:**
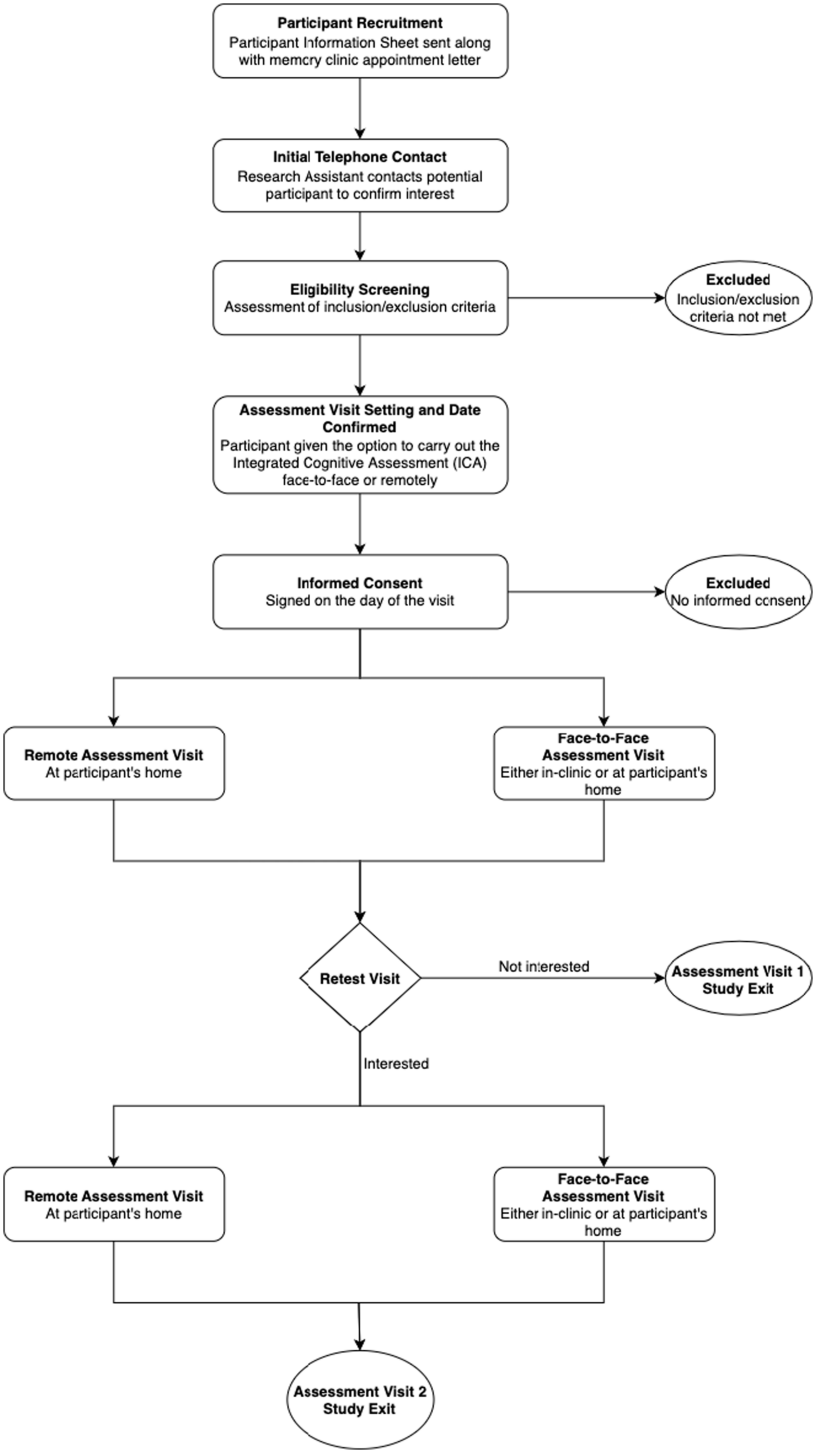
Accelerating dementia pathway technologies (ADePT) study participant pathway.

Participants were given the option to carry out a retest visit (Assessment Visit 2 [AV2]) whereby they were again given the chance to take the ICA test either remotely or face-to-face, complete a usability questionnaire, and respond to inquiries on stimulants, fatigue, and sleep. The overall study pathway for participants is detailed at a high level within Figure 2.

### Statistical analysis

The primary outcome of the study was designed to evaluate the accuracy and efficiency of GP referrals in comparison to the specialist diagnosis of MCI/dementia. Here’s a step-by-step breakdown of how this was achieved:

1. GP referrals: We began by analyzing the total number of patients referred by GPs to memory clinics. These referrals were based on the GPs’ clinical judgment of cognitive impairment.
2. Specialist diagnosis: After the GP referral, patients underwent a comprehensive assessment at the memory clinic. This assessment led to a specialist diagnosis, which could be MCI, dementia, or other conditions.
3. Comparison: We then compared the GP referrals with the specialist diagnoses. Specifically, we looked at:
  - The number of patients the GP referred to memory clinics who were later diagnosed with MCI/dementia by specialists.
  - The number of patients referred by the GP who were found not to have MCI/dementia upon specialist assessment.
4. ICA assessment: Concurrently, the same patients were assessed using the Integrated Cognitive Assessment (ICA). We then compared the ICA’s identification of cognitive impairment with the specialist’s diagnosis.
5. Outcome metrics: Based on the above comparisons, we calculated several metrics:
  - Proportion of necessary GP referrals: This is the percentage of patients referred by the GP who were later confirmed by specialists to have MCI/dementia.
  - Proportion of unnecessary GP referrals: This is the percentage of patients referred by the GP but were found not to have MCI/dementia upon specialist assessment.
  - ICA’s accuracy: We evaluated how accurately the ICA identified cognitive impairment in comparison to the specialist’s diagnosis.

By comparing GP referrals, ICA assessments, and specialist diagnoses, we aimed to demonstrate the potential of the ICA in reducing unnecessary referrals and improving the efficiency of the dementia care pathway.

For the purposes of these analyses, patients referred to the memory clinic were divided into the following 3 groups, based on their memory clinic outcome: (A) those who receive a diagnosis of MCI or dementia, (B) those who are identified as healthy or receive a diagnosis of a brain or mental disorder other than MCI or dementia, and (C) those who receive an inconclusive diagnosis.

Participants with an inconclusive outcome after the memory clinic assessment were excluded from further analysis.

Participants in group A were counted as correct GP referrals. Participants in group B were counted as unnecessary or incorrect referrals.

Comparison with Specialist Diagnosis of MCI/Dementia: The metrics for GP referrals that were calculated are the following:

- Total number of patients referred by GPs = A + B + C
- Proportion of necessary GP referrals (excluding inconclusive) = A/(A + B) [i.e., True Positive Rate]
- Proportion of unnecessary GP referrals (excluding inconclusive) = B/(A + B) [i.e., False Positive Rate]

Likewise, the following complementary metrics for the ICA were calculated:

- Total number of patients the ICA would have referred
- Proportion of patients correctly referred by the ICA (True Positive Rate)
- Proportion of patients incorrectly referred by the ICA (False Positive Rate)
- Proportion of patients correctly not referred by the ICA (True Negative Rate)
- Proportion of patients incorrectly not referred by the ICA (False Negative Rate)

In a secondary outcome analysis, we compared with specialist diagnosis of all types of cognitive impairment (those due to MCI, dementia, or other neurological or mental disorders).

### Comparison with other cognitive tests

Cut-offs were applied to cognitive tests taken in primary care and secondary care, to investigate the predictive accuracy of these tests compared to the clinical diagnosis determined at the memory clinic. Cognitive tests taken in the 6 months prior to the memory clinic assessment were used for the analysis. The results from the cognitive tests are compared to ICA results on the same patients. The cognitive tests used for this analysis were ACE-III, MoCA, MoCA Blind, GPCOG and SCIT. A sufficient number of participants had completed these assessments within 6 months of the memory clinic visit to make the analysis viable.

### Test-retest analysis

The test–retest reliability of the ICA was analyzed by the following:

- Calculation of intraclass correlation coefficient to assess test–retest reliability across all participants
- Scatterplot construction and calculation of correlation coefficient between the initial and final assessment for all participants
- Construction of Bland–Altman plots for the initial and final assessment to assess agreement

In addition to the above, we also employed the Kappa statistic (κ) to assess the test–retest reliability for the ICA as well as ICA association with the outcome of other cognitive tests (Altman, 1991). This metric provides a measure of agreement between two raters (in this case, two tests) while accounting for the agreement that might occur by chance.

The formula for the Kappa statistic is given by:


$$\mathrm{Kappa}\left(\kappa \right)=\left(Po-Pe\right)/\left(1-Pe\right)$$

Where:

- *P_o_* represents the observed proportion of agreement, calculated as the number of cases where both tests agreed divided by the total number of cases.
- *P_e_* is the expected proportion of agreement under the assumption of independence, calculated by multiplying the marginal probabilities for each rater and then summing these products.

A Kappa value of 1 indicates perfect agreement, while a value of 0 indicates agreement equivalent to chance. Interpretation of the Kappa statistic typically follows these guidelines:

- 0.00–0.20: Slight agreement
- 0.21–0.40: Fair agreement
- 0.41–0.60: Moderate agreement
- 0.61–0.80: Substantial agreement
- 0.81–1.00: Almost perfect agreement.

#### Statistical tests and significance level

We have elaborated on the specific statistical tests used throughout the text. Specifically, we employed the *t*-test or paired *t*-test where applicable, such as in the test–retest analysis. The significance level for all tests was set at *α* = 0.05 unless otherwise specified. More specifically, we made sure to include the exact value of *p*s within the manuscript for better interpretation.

#### Software and packages

The statistical analyses were conducted using Python. Specifically, we employed widely-used packages such as pandas for data manipulation, numpy for numerical computations, and scipy. Stats for statistical tests.

### Qualitative data from usability questionnaire

Multiple choice responses from participants were analyzed by calculating the proportion of participants who selected each option. Questions relating to frequency of tablet or mobile phone use were used to assess familiarity with technology, in particular, touch screen devices. The ease of understanding the ICA instructions and level of difficulty of the categorization task was analyzed by calculating the proportion of participants who reported finding each of these steps very easy, easy, moderately difficult, difficult, or very difficult.

### Procedures to account for missing and spurious data

Patients with inconclusive outcomes were excluded from our analysis. Other than that we did not have any other missing data regarding the calculations needed for primary and secondary outcome measures.

### Objectives of the ADePT study

1. *Clinical Practice Context*: Currently, GPs’ clinical judgment is the primary determinant for initiating full evaluations at memory clinics for conditions like dementia. The cognitive tests available in primary care are either rudimentary, time-consuming, or detect cognitive impairment only when the disease has advanced considerably.
2. *Introduction of the Integrated Cognitive Assessment (ICA)*: The ICA is a swift, user-friendly cognitive performance test administered on an iPad. It presents users with a series of images in quick succession, asking them to identify whether each image contains an animal. The accuracy and speed of responses are then assessed using Artificial Intelligence, comparing the results with previous ICA tests taken by both healthy and cognitively impaired individuals. This allows the ICA to offer an objective measure of cognitive performance and the likelihood of impairment.
3. *Study Aim*: The primary aim of the ADePT study was to build economic evidence, and to the wealth of the clinical data to support the adoption of the ICA as an affordable dementia screening tool within primary care settings. The study sought to determine if the ICA could enhance the dementia care pathway by refining the referral process from primary care to specialized memory clinics.

## Results

### Participants

Out of 99 participants, 86 participants completed all assessments and questionnaires successfully. Some participants did not complete the ICA (13.13%), the majority of these were patients diagnosed with dementia (69.2%), followed by 7.7% of patients with MCI, 7.7% inconclusive, 7.7% with non-dementia conditions and 7.7% healthy. Of those who did not complete the trial, 11 did not complete the ICA, either due to inability to complete the test, or due to the Researcher’s decision to cancel the test while 1 participant decided to not proceed with the study procedures. Finally there was also 1 participant that was discontinued due to passing away prior to receiving their diagnosis. Table 1 illustrates the recruited participants breakdown.

**Table 1:** Recruited participants by diagnosis following reclassification.

Diagnosis	Completed ICA			Did not complete			Overall	
	Face to Face	Remote	Total	Face to Face	Remote	Total	Overall total	Percent completed
Dementia	35	10	45	8	1	9	54	83.3
MCI	13	5	18	1	0	1	19	94.7
Healthy	10	5	15	1	0	1	16	93.3
Inconclusive	4	1	5	1	0	1	6	85.7
Non-dementia condition	3	0	3	1	0	1	4	75.0
Total	65	21	86	12	1	13	99	86.9

Recruitment is broken down by face to face and remote visits, and whether or not the participant completed all assessments including the ICA.

The distribution of age and education years for all participants is shown in Table 2, and broken down by diagnosis in Table 3.

**Table 2:** Age and education years of all participants.

Age							Education years						
*N*	Mean	Std	Median	Min	Max	Range	*N*	Mean	Std	Median	Min	Max	Range
87	76.0	8.1	77.5	56	89	33	86	13.8	3.9	12	10	30	20

**Table 3:** Age and education years of participants per diagnosis.

Diagnosis	Age							Education years						
	*N*	Mean	Std	Median	Min	Max	Range	*N*	Mean	Std	Median	Min	Max	Range
Dementia	45	79.1	6.9	79	63	89	26	45	13.2	3.5	12	10	24	14
MCI^*^	18	74.7	5.5	74.5	64	82	18	18	13.8	3.3	13	10	20	10
Healthy	14	69.2	8.9	68.5	57	85	28	14	15.7	5.6	15	11	30	19
Inconclusive	6	70.7	11.3	72.5	56	85	29	6	14.8	4.4	13.5	11	23	12
Non-dementia condition	3	78.3	5.5	81	72	82	10	3	12.3	2.5	12	10	15	5

^*^MCI, Mild Cognitive Impairment.

### Comparison of GP/ICA referrals with specialist diagnosis

The following analysis is based on participants who completed the required assessments and questionnaires.

### Comparison of GP/ICA referral with specialist diagnosis of MCI/dementia

Of the 86 participants who completed the ICA, 5 had inconclusive diagnosis, hence the number of participants under investigation for the primary outcome analysis is 81 (Tables 4–7).

**Table 4:** Comparison of precision of GP referrals with specialist diagnosis of MCI/dementia.

Total referred by GP	86
Total referred by GP excluding inconclusive	81
Those with diagnosis of MCI or dementia	63
Those who are healthy or with diagnosis of disorders other than MCI/dementia	18
Inconclusive diagnosis	5
Proportion of necessary referrals by GP	77.8%
Proportion of unnecessary referrals by GP	22.2%

**Table 5:** GP classification matrix.

		GP referral	
		Not referred	Referred
Specialist diagnosis	Healthy/Other	NA	18
	MCI/Dementia	NA	63

**Table 6:** AI V1 classification matrix.

		ICA AI V1	
		Healthy	Impaired
Specialist diagnosis	Healthy/Other	13	5
	MCI/Dementia	6	57

**Table 7:** Comparison of ICA referral with specialist diagnosis of MCI/dementia.

	Primary outcome analysis
	AI V1
Total ICA would have referred	62
Proportion correctly referred by ICA	90.5%
Proportion incorrectly not referred by ICA	9.5%
Proportion correctly not referred by ICA	72.2%
Proportion incorrectly referred by ICA	27.8%

### Comparison of GP/ICA precision in referral with specialist diagnosis of all types of impairment

In this analysis the precision of GP referral is compared to specialist diagnosis of MCI, dementia and all other types of non-dementia neurological/mental disorders.

Tables 8, 9 provide a comprehensive breakdown of GP referrals in relation to specialist diagnoses for various cognitive impairments. They detail the total number of patients referred, those diagnosed with specific conditions, and the proportions of necessary and unnecessary referrals. Overall, 81.5% of the GP referrals were necessary, aligning with specialist diagnoses of cognitive impairments, while 18.5% were deemed unnecessary.

**Table 8:** Comparison of GP referral precision with specialist diagnosis of all types of impairment.

Total referred by GP	86
Total referred by GP excluding inconclusive	81
Those with diagnosis of MCI or dementia or other neurological/mental disorders	66
Those who are healthy	15
Inconclusive diagnosis	5
Proportion of necessary referrals by GP	81.5%
Proportion of unnecessary referrals by GP	18.5%

**Table 9:** GP classification matrix.

		GP referral	
		Not referred	Referred
Specialist diagnosis	Healthy	NA	15
	MCI/Dementia/Other	NA	66

Tables 10, 11 provide insights into the performance of the ICA test in classifying cognitive impairments. The ICA AI outcome demonstrated an accuracy of 0.87 *[CI*^1^*: 0.81, 0.94]* with a commendable AUC of 0.93 *[CI: 0.86, 0.99]*. Specifically, the sensitivity and specificity in detecting cognitive impairment were 0.89% *[CI: 0.82, 0.96]* and 0.80 *[CI: 0.61, 0.97]*, respectively. A detailed breakdown of sensitivity and specificity for MCI and dementia, separately, as well as a comparison to the standard of care pen and paper tests, is further elucidated in Table 12. When comparing these metrics to the GP referrals from Table 8, the ICA shows a higher accuracy in correctly identifying patients with cognitive impairments. The ICA would have referred 62 patients, with 89.4% of these referrals being correct, further emphasizing its potential as a reliable screening and referral tool.

**Table 10:** AI V1 classification matrix and performance metrics (healthy vs. impaired).

		ICA AI V1	
		Healthy	Impaired
Specialist diagnosis	Healthy	12	3
	MCI/Dementia/Other	7	59

ICA AI V1 performance metrics: Accuracy = 87.6; AUC = 93.0; Sensitivity = 89.4; Specificity = 80.0.

**Table 11:** Comparison of ICA referral with specialist diagnosis of all types of impairment.

	Secondary outcome analysis
	AI V1
Total ICA would have referred	62
Proportion correctly referred by ICA	89.4%
Proportion incorrectly not referred by ICA	10.6%
Proportion correctly not referred by ICA	80.0%
Proportion incorrectly referred by ICA	20.0%

**Table 12:** Performances by ICA and standard care tests (meta-data) at glance.

	MCI		Dementia	
	Sensitivity	Specificity	Sensitivity	Specificity
ICA tool (CognICA)	83%	80%	93%	80%

Standard care test^*^	MCI		Dementia		Proportion of patients receiving
	Sensitivity	Specificity	Sensitivity	Specificity	
Mini-Mental State Examination (MMSE)	51%	75%	59%	85%	26%
General Practitioner Assessment of Cognition (GPCOG)	52%	82%	60%	93%	21%
6-Item Cognitive Impairment Test (6CIT)	66%	70%	88%	78%	29%
Test Your Memory (TYM)	NA^**^	NA	93%	86%	0%
Memory Impairments Screen (MIS)	NA	NA	80%	91%	0%
Abbreviated mental test score (AMTS)	66%	70%	81%	84%	7%
Montreal Cognitive Assessment (MoCA)	80%	81%	91%	81%	6%
Other	63%	76%	79%	85%	11%
Weighted	60%	75%	73%	84%	

^*^See Shore et al. (2022) for a summary of relevant literature reporting on standard care tests. ^**^NA, Not available.

Note that in Tables 9, 10 the reported performance metrics for the classification is healthy vs. impaired (i.e., MCI/Dementia/Other). The breakdown of sensitivity and specificity for healthy vs. MCI and healthy vs. dementia is included in Table 12.

### Test-retest analysis

**Figure 3:**
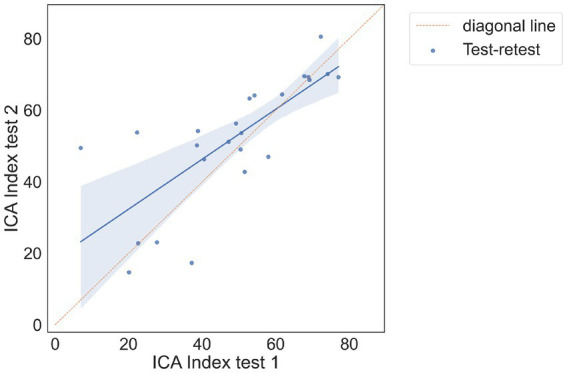
Correlation plot between test and retest, showing a strong positive correlation between the two tests.

**Figure 4:**
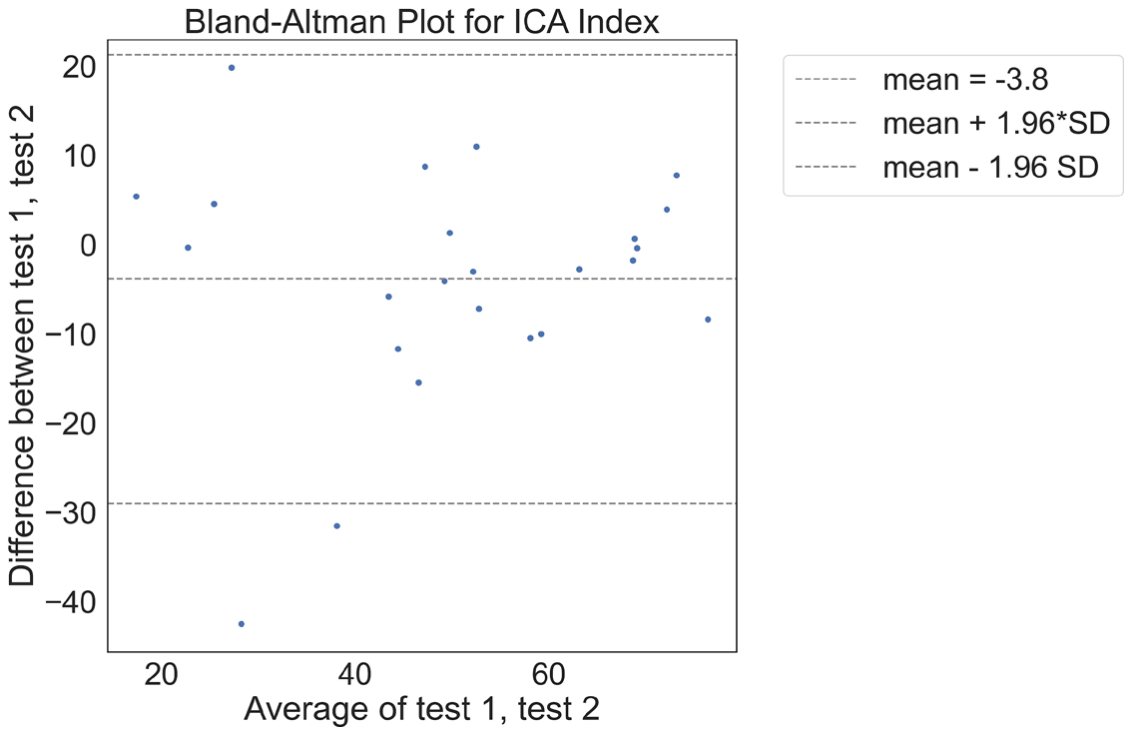
Bland Altman plot of differences between test and retest.

There were 24 participants who completed the initial visit and the retest. On average, there were 9.7 days between the test and retest (with an SD of 3 days, ranging from minimum of 5 days to a maximum of 14 days). The correlation between the ICA test retest is shown in Figure 3. The Spearman Rank coefficient for the test–retest ICA Index was calculated to be 0.79 (*p* < 0.000). While the average ICA Index in the retest group was 3.8 points higher than the initial test (see Bland Altman plot in Figure 4), paired *t*-test value of *p* was >0.1, suggesting this was not a statistically significant increase in score on the retest (Table 13). The Spearman Rank coefficient for the test–retest of ICA AI score was found to be 0.81 (*p* < 0.000).

**Table 13:** Correlation coefficients and other metrics associated with test–retest.

Metric	ICA index
Pearson *r*	0.75
Pearson value of *p*	0.00
Spearman *r*	0.79
Spearman value of *p*	0.00
ICC	0.738 [0.49, 0.88]
*t*-test statistic	−1.43
*t*-test value of *p*	0.17
Cohen’s D	−0.21
Mean test 1	48.32
Mean test 2	52.14
Difference test1-test2	−3.81
SD test 1	18.59
SD test 2	17.35

The 2×2 matrix of ICA prediction on the first and second test is shown in Table 14.

**Table 14:** ICA prediction on first and second visit.

		ICA prediction visit 2	
		Healthy	Impaired
ICA prediction visit 1	Healthy	7	0
	Impaired	3	14

The test-retest prediction agreement for the ICA is 87.5%.

In assessing the consistency of the ICA predictions between the first and second visits, we also utilized the kappa statistic (κ) to measure the agreement while accounting for chance agreement (Altman, 1991). Typically, Kappa values of 0.41 to 0.60 are considered moderate and a kappa of 0.61 to 0.80 represents substantial agreement. The observed proportion of agreement between the two visits was 87.5%. After calculations, the kappa statistic was found to be 0.7427. This value suggests a substantial level of agreement between the ICA predictions on the initial visit and the subsequent visit. This high degree of consistency underscores the reliability of the ICA tool across repeated assessments.”

### Comparison of cognitive test cut-offs with ICA

In this section cut-offs are applied to cognitive tests taken in primary care and secondary care, to investigate the predictive accuracy of these tests compared to the clinical diagnosis determined at the memory clinic. Cognitive tests taken in the 6 months prior to the memory clinic assessment were used for the analysis. The results from the cognitive tests are compared to ICA results on the same patients.

The cognitive tests used for this analysis are ACE-III, MoCA, MoCA Blind, GPCOG and SCIT. A sufficient number of participants had completed these assessments within 6 months of the memory clinic visit to make the analysis viable.

We also calculated the correlation between the ICA Index and cognitive tests taken in memory clinics and GP settings, where there was at most 12 months difference in date between the tests. Results were included where there were at least 10 data points for the correlation calculation. The ICA index had a Pearson correlation of 0.57 (*p* < 0.000) with ACE-III and 0.77 (*p* < 0.002) with MoCA, both of which were taken as part of the memory clinic assessment. The ICA had a lower correlation with the more rudimentary assessments taken in the GP setting, e.g., a correlation of 0.37 (*p* < 0.18) with GPCOG (see Appendix A1 for more details).

### ACE-III memory clinic assessment

The ACE-III was the most common memory clinic assessment, completed by 68 participants. A cut off score of ≥88 was used for ACE-III scores for binary prediction of healthy and impaired. On average the ACE and ICA assessments were conducted 38 days and 24 days from the diagnosis date, respectively.

ACE outcomes for participants, broken down by diagnosis, are shown in Table 15, and compared with ICA outcomes for the same patients. A comparison of these cognitive tests should also consider that the average ICA completion time is ~9 min, compared to approximately 30 min for ACE-III.

**Table 15:** ACE and ICA labels compared to the clinical diagnosis.

Clinical diagnosis	Total	ACE		ICA		
		Healthy	Impaired	Healthy	Impaired	Did not complete
Dementia	37	1	36	3	30	4
MCI	14	5	9	2	12	0
Inconclusive	2	0	2	0	1	0
Healthy	12	12	0	9	2	1
Non-dementia condition	3	2	1	0	2	1
Total	68	20	48	14	47	6

Excluding inconclusive cases, the overall accuracy of ACE-III on these participants (*n* = 66) is 88%. Here we assumed that detecting cognitive impairment in non-dementia cases is a correct classification.

Comparing ICA and ACE predictions, where both the ICA and ACE were completed, results in the matrix shown in Table 16.

**Table 16:** ICA and ACE prediction comparison in 2×2 matrix.

		ICA prediction	
		Healthy	Impaired
ACE prediction	Healthy	10	9
	Impaired	4	38

The overall percent agreement between the ICA and ACE is 79%. The kappa statistic for ICA vs. ACE is 0.464, indicating moderate agreement. Supplementary Table S3 shows the diagnosis, ICA and ACE scores for the participants where the ICA and ACE had differing predictions.

### MoCA memory clinic assessment

For MoCA assessments, which were conducted in memory clinics, a cut-off of 26 or greater was used to predict healthy patients. On average the ICA test was taken 17 days apart from the diagnosis date, while the MoCA assessment was taken 58 days from diagnosis. MoCA outcomes for participants, broken down by diagnosis, are shown in Table 17.

**Table 17:** Comparison of predictions from MoCA and ICA.

Diagnosis	Total	MoCA_Healthy	MoCA_Impaired	ICA_did_not_complete	ICA_Healthy	ICA_Impaired
Dementia	3	0	3	2	0	1
Healthy	3	3	0	0	2	1
MCI	3	2	1	0	1	2
Inconclusive	2	1	1	0	0	2
Non-dementia condition	1	1	0	0	1	0
Totals	12	7	5	2	4	6

Excluding inconclusive cases and non-dementia conditions, the overall accuracy of MoCA on these participants (*n* = 10) is 70%. Here we assumed that detecting cognitive impairment in non-dementia cases is a correct classification.

Comparing ICA and MoCA predictions, where both the ICA and MoCA were completed, results in the matrix shown in Table 18.

**Table 18:** 2×2 comparison of MoCA and ICA predictions.

		ICA prediction	
		Healthy	Impaired
MoCA prediction	Healthy	4	3
	Impaired	0	3

Overall the ICA and MoCA have a percentage agreement of 70%. The kappa statistic for ICA vs. MoCA is 0.444, suggesting a moderate agreement. The cases where the ICA and MoCA prediction were different are shown in Supplementary Table S4.

### MoCA blind memory clinic assessment

This analysis was based on MoCA Blind assessments carried out by the memory clinic. A score ≥ 18 for healthy was used as the cut-off for healthy participants (Mahendran et al., 2015). The ICA assessment was 15 days from diagnosis date, while the MoCA Blind assessment was 16 days from diagnosis date. MoCA Blind outcomes for participants, broken down by diagnosis, are shown in Table 19.

**Table 19:** Comparison of predictions from MoCA Blind and ICA.

Diagnosis	Total	MoCA_blind_Healthy	MoCA_blind_Impaired	ICA_did_not_complete	ICA_Healthy	ICA_Impaired
Dementia	13	0	13	2	0	11
MCI	1	1	0	0	0	1
Totals	14	1	13	2	0	12

The overall accuracy of MoCA Blind for these participants (*n* = 14) is 93%. Note the small sample size in comparison to ICA and ACE. Here we assumed that detecting cognitive impairment in non-dementia cases is a correct classification.

Comparing ICA and MoCA Blind predictions, where both the ICA and MoCA Blind were completed, results in the matrix shown in Table 20.

**Table 20:** 2×2 comparison of MoCA Blind and ICA predictions.

		ICA prediction	
		Healthy	Impaired
MoCA Blind prediction	Healthy	0	1
	Impaired	0	11

The kappa statistic for the comparison between ICA and MoCA Blind is 0, indicating no agreement beyond chance, despite a high observed agreement of 92%. This discrepancy is due to the skewed distribution in the contingency table and the small sample size.

There was only one instance (from 12) in which the assessments differed (Supplementary Table S5). In this case the ICA predicted impairment, in agreement with the clinical diagnosis of MCI, while the MoCA Blind score of 18 was suggestive of a healthy patient.

### GPCOG in primary care assessment

The following cut offs were used for the GPCOG assessment, based on Brodaty et al. (2004):

- Score of 0–4: cognitive impairment indicated
- Score of 5–8: more information required
- Score of 9: no significant impairment (for this case we have indicated Healthy for direct comparison with the ICA)

The ICA test was completed on average 15 days before diagnosis, while the GPCOG test was completed on average 67 days before diagnosis.

GPCOG outcomes for participants, broken down by diagnosis, are shown in Table 21.

**Table 21:** Comparison of predictions from GPCOG and ICA.

Clinical_diagnosis_short	Total	GPCOG_Further Investigate	GPCOG_Healthy	GPCOG_Impaired	ICA_did_not_complete	ICA_Healthy	ICA_Impaired
Dementia	12	4	3	5	3	2	7
Healthy	3	0	3	0	0	2	1
MCI	1	0	1	0	0	0	1
Totals	16	4	7	5	3	4	9

The overall accuracy of GPCOG for these participants (*n* = 16) is 75%. Note the small sample size in comparison to ICA and ACE. Here we assumed that detecting cognitive impairment in non-dementia cases is a correct classification. We also combined ‘Further Investigate’ with ‘Impaired’ to indicate that GPCOG had identified cognitive impairment.

Comparing ICA and GPCOG predictions, where both the ICA and GPCOG were completed, results in the matrix shown in Table 22.

**Table 22:** Comparison matrix of GPCOG and ICA predictions.

		ICA prediction	
		Healthy	Impaired
GPCOG prediction	Further Investigate	2	1
	Healthy	2	5
	Impaired	0	3

The observed percent agreement between GPCOG and ICA is 0.50 (excluding cases labeled as ‘Further Investigate’). The kappa statistic for the comparison between ICA and GPCOG is 0.193, suggesting only a slight agreement beyond chance.

### 6CIT in primary care assessment

For the 6CIT assessment, carried out at GPs, a cut off score of 8 or greater was used to indicate cognitive impairment, based on Brooke and Bullock (1999). For the participants who completed the 6CIT and ICA, the ICA was completed on average 31 days before diagnosis, while the 6CIT test was completed on average 103 days before diagnosis.

6CIT outcomes for participants, broken down by diagnosis, are shown in Table 23.

**Table 23:** Comparison of predictions from 6CIT and ICA.

Diagnosis	Total	SCIT_Healthy	SCIT_Impaired	ICA_Healthy	ICA_Impaired
Dementia	11	3	8	1	9
Healthy	2	1	1	2	0
Inconclusive	1	1	0	0	1
Totals	14	5	9	3	10

Excluding inconclusive cases, the overall accuracy of 6CIT for these participants (*n* = 13) is 69%.

Comparing ICA and 6CIT predictions, where both the ICA and 6CIT were completed, results in the matrix shown in Table 24.

**Table 24:** 2×2 comparison of 6CIT and ICA predictions.

		ICA prediction	
		Healthy	Impaired
SCIT prediction	Healthy	1	4
	Impaired	2	6

The observed percent agreement between 6CIT and ICA is 0.54. The kappa statistic for the comparison between ICA and 6CIT is −0.05, suggesting that there is no agreement between ICA and 6CIT beyond what would be expected by chance.

### Sensitivity and specificity of the ICA test versus standard care tests

Table 12 provides a comprehensive comparison of the ICA tool with various standard care cognitive tests in terms of their sensitivity and specificity for detecting cognitive impairment in MCI and Dementia. The table showcases the performance metrics of the ICA alongside several widely recognized cognitive tests, offering a clear perspective on how the ICA stands in relation to these standard assessments.

For MCI, the ICA tool demonstrates a sensitivity of 83% and a specificity of 80%. For Dementia, its sensitivity rises to 93%, while maintaining a specificity of 80%. These figures are juxtaposed with the metrics from standard care tests, such as the MMSE, GPCOG, 6CIT, and others. The table also provides the proportion of patients receiving each of these standard tests, offering insights into their prevalence in clinical settings.

The weighted row at the bottom of the table gives a consolidated view, factoring in the proportion of patients for each test, of the average sensitivity and specificity across all standard care tests. This provides a benchmark against which CognICA’s performance can be evaluated.

In comparison to the weighted average of standard care tests, CognICA demonstrates superior sensitivity for both MCI and Dementia, while maintaining comparable specificity.

## ICA usability

### ICA completion rate

An update to the CognICA application introduced a new test flow, which automated the pre-test demo/trial image process. More specifically, the pre-test image process was automated while a training video was introduced to facilitate remote assessments. These changes did not affect the core of the test.

This resulted in an increased ICA completion rate among the groups with impaired diagnoses (Table 25). For example for dementia patients the completion rate increased from 78 to 89%.

**Table 25:** Completion rate of the ICA, across diagnoses, based on the cognICA version.

Diagnosis	CognICA version 1.2.1				CognICA version 1.3.2				Overall total
	Completed	Did not complete	Completion (%)	Total	Completed	Did not complete	Completion (%)	Total	
Dementia	21	6	77.8	27	24	3	88.9	27	54
MCI	6	1	85.7	7	12	0	100.0	12	19
Healthy	5	0	100.0	5	10	1	90.9	11	16
Inconclusive	2	0	100.0	2	4	0	100.0	4	6

A further benefit of the updated test flow was a reduction in the number of trial images completed by patients. The completion rate of the ICA could also have been influenced by the new training video. This was introduced partway through the study, and is discussed in more detail further on. However, among dementia patients, there was an increased rate of completion (from 80 to 85%) when the video was watched compared to when it was not watched.

### ICA completion time

On average, from device hand over to completion of the test the ICA took ~9 min to complete in this setting. This is based on the researcher’s approximation of the time taken, and is not a precise measurement. The mean time taken for ICA completion was reduced from visit 1 to visit 2, from 9 min to 7 min (Supplementary Table S9) face-to-face administrations were on average 1 min shorter than remote administrations of the test (Supplementary Table S10); and across diagnoses, healthy individuals completed the test 3 min faster than cases with a diagnosis of dementia (Supplementary Table S11).

The *t*-test of visit 1 to visit 2 time, *p* = 0.03. This suggests that as expected the second test takes less time to complete compared to the first test. The *t*-test of face to face vs. remote test time taken *p* = 0.16, suggesting there is no significant difference in time taken across the two scenarios. While the meantime taken of all tests is less than 10 min, all cases where the time taken was >15 min occurred for dementia patients.

### Tablet use

Of all the participants who completed the questionnaire (*n* = 99), about 41% had no prior experience with tablets, while ~35% used tablets on a daily or frequent basis, the rest used tablets only a few times a week (%10) or otherwise very rarely (~14%).

Healthy participants were more likely to be frequent users of tablets compared to MCI and Dementia patients. It is important to consider here that the sample size for healthy patients is smaller (*n* = 15) than MCI patients (*n* = 19), and Dementia patients (*n* = 54) (Supplementary Figure S1).

### Smartphone use

There is a higher percentage of regular smartphone usage (~%45) –compared to tablet–, however a similar pattern exists suggesting that those with higher levels of cognitive impairment use smartphone devices on a less regular basis (Supplementary Figure S2).

### ICA remote operation

Of the 22 participants who attempted the ICA remotely for their first visit, only one chose not to continue and did not complete the ICA. The majority were able to operate the ICA remotely without difficulty. 55% of participants found it very easy or easy to operate the device from receiving the device to starting the instructions. 19% rated the experience with medium difficulty.

Those with some previous tablet experience found it easier to operate the device compared to those who had never used tablets previously.

In the retest (7 participants conducted a remote retest), none of the participants indicated a difficult/very difficult experience in operating the ICA remotely.

### Difficulty in understanding ICA instructions

Most participants (~68%) found it very easy or easy to understand the ICA instructions. Those with cognitive impairment (particularly dementia patients) were more likely to find understanding the ICA test instructions difficult or very difficult (Supplementary Figure S3).

Those with some tablet experience were more likely to find understanding the test instructions very easy or easy, and less likely to find it difficult. However a confounding factor is that healthy patients are more likely to use tablet devices.

Looking just at dementia patients, we find that within this subgroup the same pattern of easier understanding of instructions with increased tablet use exists.

On retest visits, in comparison to the first visit, a higher proportion of participants found the test instructions very easy or easy (80%), with a lower percentage indicating difficulty.

Most participants had no or little anxiety in taking the ICA. On self-reported anxiety levels, MCI and dementia participants were more likely to report more anxiety compared to other groups. 10% of MCI and dementia cases said they became very anxious, compared to none (0%) in other groups.

All participants who undertook a retest reported either the same or lower level of anxiety on the retest. There were no reports of increased anxiety on the retest.

### Difficulty in taking the ICA test itself

Overall most participants found the ICA test challenging, with less than 20% identifying the test as very easy or easy. This is to be expected as the test is designed to be challenging, there is no ceiling effect, and it is rare even for young healthy individuals to get a full score on the test.

However, after the retest the perception of participants changed, with the most common response being ‘medium’ difficulty (Supplementary Figure S4).

Approximately 55% of those who did a retest indicated the second test was as difficult as their first, while 46% indicated they found the second test easier.

In the majority of cases participants were able to carry out the test without distraction, whether in face to face visits (~85%) or remotely (~70%). Participants were more likely to report being ‘a little distracted’ in remote tests compared to face to face tests (21% compared to 8%).

### How did the participants hold the device?

There is variation in how participants held the iPad during the test. The most common (and recommended) position was held in both hands, however ~22% of participants placed the iPad on a stand, and ~ 32% placed the iPad flat on the table.

Participants with cognitive impairment were less likely to hold the device in both hands, and more likely to place the tablet flat on the table.

However, with some tablet use, participants are more likely to hold the device in both hands. Supplementary Figure S5 in the supplement shows this for dementia patients.

### Engagement and willingness to take the test again

A significant majority of participants (~84%) expressed willingness to take the ICA again in the future. The percentage was slightly lower for those with cognitive impairment (~78%).

The percentage of those willing to take the ICA again increased to over 90% for those who also completed the retest.

## Discussion

This study, conducted as part of the ADePT project, has provided quantitative and qualitative evidence on the effectiveness and usability of the ICA in real world clinical environments. In previous studies, such as Khaligh-Razavi et al. (2019) and Kalafatis et al. (2021), the sensitivity of the ICA in detecting cognitive impairment in MCI and mild AD patients was investigated in research and clinical-trial settings. This study focused on the comparison of the ICA classification with GP referral, and ultimately the specialist diagnosis from the memory clinics. The ICA was tested in a wider range of patients who had been referred to memory clinics, including those with more progressed dementia, those with non-dementia conditions, and a number of inconclusive cases.

### Comparison with GP referrals and memory clinics

The primary outcome of the study compared GP referral with specialist diagnosis of MCI/dementia. Of those the GP referred to memory clinics, 78% were necessary referrals, with ~22% unnecessary referrals, or patients who should have been referred to other services as they had disorders other than MCI/dementia. In the same population, the ICA was able to correctly identify cognitive impairment in ~90% of patients, with approximately 9% of patients being false negatives. From the subset of unnecessary GP referrals, the ICA classified ~72% of those as not having cognitive impairment, suggesting that these unnecessary referrals may not have been made if the ICA was in use.

The proportion of necessary GP referral in our work is higher than what has been found in other studies like Creavin et al. (2021), where 56% was reported. It is likely that the GPs’ referrals assessed for the purpose of this study had a special interest in Dementia diagnosis, thus the higher rate of accurate referrals. The sample size here is less than 100 participants from four sites in the UK, therefore GP referral accuracy from larger studies should also be considered. However the GP referral precision obtained from this study aligns with the values obtained from Sussex audit report for 2020–2021, which indicated there was no diagnosis of cognitive impairment in only 8% of cases from those who presented at the memory clinic.

Due to the challenges in recruiting patients from various GP sites, a main limitation of this study is that only those who were referred by GPs have been investigated. Therefore, the results from this study cannot be used to determine the false negative rate, i.e., those who did have cognitive impairment but were not referred by their GP. We seek to address this in future studies where the ICA will also be deployed in primary care.

### Remote administration of ICA in response to COVID-19

In response to the COVID-19 pandemic, and the move toward remote assessments, the study protocol was amended to allow remote administration of the ICA. Twenty-two participants attempted the ICA remotely, with only one participant not wishing to continue. Most patients found the remote operation of CognICA very easy or easy, however, those with no prior tablet experience were more likely to have difficulty. In the majority of cases participants were able to carry out remote tests without distraction, however participants were more likely to report slight distraction in remote tests compared to face to face tests.

### Usability and patient experience

Overall the usability results show that most participants were able to understand the ICA instructions and complete the test, even though they found the rapid visual categorization task itself challenging. Patients’ usability experience was found to be related to previous familiarity with phones and tablets; those with some prior experience found it easier to understand the instructions and to operate the device. They were also more likely to hold the device in both hands rather than placing it flat on the table. There were also differences in perception among patients based on their clinical diagnosis. As expected those with MCI, dementia or a non-dementia condition were more likely to have difficulties, and to feel slight or mild anxiety in taking the ICA.

Those who took a retest reported an improved overall usability experience. They found the instructions easier to understand, experienced less or the same level of anxiety, found the test easier or the same as before and found operating the device in remote assessments easier than the first visit. Both after the first and second visits, a significant majority of patients indicated they would be willing to take the ICA again in the future.

Some patients did not manage to complete the test, in particular those with dementia or more severe cognitive impairment. An update to the ICA software which allowed participants to seamlessly progress from the pre-test demo and trial images to the main test, as well as the introduction of a new instruction video helped to increase the completion rate. For dementia patients the increase was from 78% in CognICA 1.2 to 89% in CognICA 1.3. It is important to note that ICA is particularly designed to detect and monitor subtle cognitive impairments or changes to cognitive performance in earlier stages of the disease, where there is an unmet need in clinical practice.

### ICA adoption in primary care and integrated care systems

The Integrated Cognitive Assessment (ICA) emerges as a pivotal tool in the realm of primary care, addressing several challenges inherent in the current referral process for cognitive impairment.

The ICA’s objective measure of cognitive performance offers a potential solution to refine the referral process from primary care to specialized memory clinics. By accurately identifying those with cognitive impairment, the ICA can help ensure that those who truly require specialized care are referred, reducing unnecessary referrals, as well as reducing undiagnosed or late-diagnosis of dementia.

The economic strain on healthcare systems, particularly the NHS, is significant. By reducing the number of unwarranted referrals, the ICA not only alleviates the financial burden but also optimizes resource allocation, ensuring that specialized care is available to those who genuinely need it.

Early detection is paramount in managing cognitive impairment. The ICA facilitates this by offering a swift, non-intrusive assessment that can be easily integrated into primary care settings. This ensures that patients receive timely interventions, leading to better outcomes and an enhanced quality of life. This timely diagnosis can further lead to significant cost savings for the NHS, as highlighted by Shore et al. (2022). This becomes even more pertinent with the advent of new disease-modifying therapies for Alzheimer’s disease on the horizon.

The real-world data from the ADePT study, in conjunction with previous clinical studies of the ICA (Khaligh-Razavi et al., 2019 and Kalafatis et al., 2021), provides compelling evidence for the efficacy of the ICA as a screening tool. This robust evidence base underscores the potential of the ICA to revolutionize the dementia care pathway, starting at the primary care level.

In light of these considerations, the adoption of the ICA in primary care settings presents a forward-thinking approach to dementia care, aligning with the broader objectives of enhancing patient outcomes, optimizing resource utilization, and ensuring cost-effectiveness.

## Conclusion

The results from this study demonstrate the potential of the ICA as a screening tool, which can be used to support accurate referrals from primary care settings, along with the work conducted in memory clinics and in secondary care. The study investigated a cohort of patients aged 55–90, who were recruited at NHS memory services. The aim of the study was to gather real-world evidence of the use of the ICA in memory clinics and compare the performance of the ICA versus GP referrals.

The ICA test has proven to be a consistent method of detection of cognitive impairment, with an initial test and a retest being strongly, positively correlated (*r* = 0.88, *p* < 0.000) and a test retest prediction agreement of 87.5%. Furthermore, the ICA and more traditional tests, like ACE-III or MoCA, share a percent agreement in the range of 70–90%. Analysis demonstrated that, if adopted, the ICA would have reduced unnecessary referrals from GPs. Paired with a faster delivery time (approximately 5–10 min, compared to about 30 min for a traditional test like ACE-III), the ICA can prove an effective tool in the assessment of cognitive impairment (Shore et al., 2022).

Most participants across clinical diagnoses could complete the test and understand the ICA instructions, even though about half of the cohort would very rarely or never use a tablet. A third of the cohort found the test challenging, but patients’ usability experience improved during the retest. Furthermore, the introduction of an updated version of the cognICA software (with pre-test demo and trial images available to users) has proved to increment user engagement and completion rate, demonstrating the potential of this tool.

By comparing the precision of GP referrals against the ICA, it becomes evident that the ICA offers a more efficient and cost-effective model. The higher precision of the ICA translates to fewer unnecessary referrals, leading to cost savings and better resource allocation. From a patient perspective, the ICA ensures a smoother and more accurate diagnostic journey.

Adopting the ICA as a screening tool in primary care settings aligns with the objectives of enhancing patient outcomes, optimizing resource utilization, and ensuring cost-effectiveness in the dementia care pathway.

## Data availability statement

The original contributions presented in the study are included in the article/Supplementary material, further inquiries can be directed to the corresponding author.

## Ethics statement

The studies involving humans were approved by Approved 27/02/2020, North of Scotland Research Ethics Committee (Summerfield House, 2 Eday Road, Aberdeen, AB15 6RE, UK; +44 (0)1224 558,458; nosres@nhs.net), ref.: 20/NS/0029. The studies were conducted in accordance with the local legislation and institutional requirements. The participants provided their written informed consent to participate in this study.

## Author contributions

CK, NT, and S-MK-R designed the study. MM analyzed the data. MM, PA, CK, NT, and S-MK-R wrote the manuscript. All authors contributed to the article and approved the submitted version.

## Funding

This study was funded by Innovate UK in form of a grant to Cognetivity Ltd. (#105837).

## Conflict of interest

CK is a shareholder at Cognetivity Ltd. S-MK-R is a CIO and shareholder at Cognetivity Ltd. MM and PA are previous employee and stock options holder at Cognetivity Ltd.

The remaining author declares that the research was conducted in the absence of any commercial or financial relationships that could be construed as a potential conflict of interest.

## Publisher’s note

All claims expressed in this article are solely those of the authors and do not necessarily represent those of their affiliated organizations, or those of the publisher, the editors and the reviewers. Any product that may be evaluated in this article, or claim that may be made by its manufacturer, is not guaranteed or endorsed by the publisher.

## Footnotes

^1^95% confidence interval.
